# Densely packed membrane configurations

**DOI:** 10.1007/s11012-024-01923-x

**Published:** 2024-12-17

**Authors:** Stefanie Heyden, Michael Ortiz

**Affiliations:** 1 ETH Zurich, 8092 Zurich, Switzerland; 2 California Institute of Technology, Pasadena, CA 91125 USA; 3 Universität Bonn, 53115 Bonn, Germany

**Keywords:** Fluid membranes, Dense packings, Director field method

## Abstract

We put forth a simple mathematical model of densely packed fluid membranes and solve for packing configurations that minimize their elastic energy. Numerical calculations are facilitated via a finite-difference discretization scheme. Absent topological constraints, energy-minimizing configurations are found to closely follow solutions of the eikonal equation. These typically involve foliations comprising many closed surfaces. We show how allowing for cuts and creases, with an additional minimization over the total crease energy, generates configurations consisting of a densely packed single sheet.

## Introduction

In which configuration does a folded membrane minimize its elastic energy? We encounter this question in numerous settings, ranging from cell organelles and folding inside buds [1], to self-folding devices in biomedical applications [2] and compactly folded membranes in deployable structures [3]. In most cases, maximizing the amount of folded membrane in a given volume is favorable—not only as a design premise in engineering applications, but also as developed by nature itself. This tradeoff can easily be seen in cells with high energy demand: The mitochondrion in cardiomyocytes for example exhibits an inner membrane area that is tenfold that of its outer barrier membrane [4].

The *crumpling* of paper, as a model problem for the behavior of confined thin sheets, has received the attention of numerous authors [5–11] in the classical physics literature. That work for the most part focused on specific types of canonical singularities and, on the basis of heuristic arguments, led to the conjecture that the elastic energy per unit thickness of a crumpled membrane scales as $h^{5/3}$ for small thickness *h*. The problem has also elicited considerable interest in the mathematical literature (cf., e.g., [12] for a review).

The majority of computational studies on folded membranes relies on a discrete membrane representation (see, e.g., [13–16]). These studies allow for the resolution of intricate phenomena during membrane folding, such as membrane/protein and membrane/particle interactions [17–20]. In the realm of folded membrane configurations, they have furthermore been applied to predicting the shapes of fluid membrane vesicles in confinement [21]. However, discrete membrane representations based on thin shell theory are typically costly and technically complex. They furthermore fall short at describing settings in which membrane topologies either rearrange, or in which they are a priori unknown.

For densely packed configurations, continuum models based on a *director field* theory can be advantageous. Director field theories have been explored, e.g., in the realm of DNA packaging [22] and membrane fusion [23]. In this work, we put forth a simple mathematical model of densely packed fluid membranes and search for packing configurations that minimize their elastic energy. To enable numerical calculations and experiments, a finite-difference discretization scheme is also formulated that is remarkable for its simplicity of implementation and efficiency. Absent topological constraints, energy-minimizing configurations are found to typically involve foliations comprising many closed surfaces. We show how allowing for cuts and creases, with an additional minimization over the total crease energy, generates configurations corresponding to one single densely packed sheet.

## Director field formulation

**Fig. 1:**
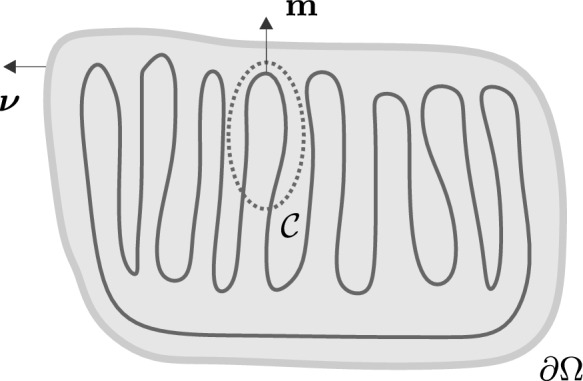
Packed membrane configuration in confinement, illustrating director field $\textbf{m}$, outward unit normal $\nu$, rigid confinement boundary $\partial \Omega$, and exemplary closed loop *C*

A simple model of packed membranes can be formulated by means of a *director field* $m:\mathbb {R}\rightarrow S^2$ such that *m*(*x*) is the normal to the membrane at *x*, as illustrated in Fig. 1. For simplicity, we shall assume that the area of membrane per unit volume takes a constant value $\sigma$. We shall further assume that the membrane is continuous. This condition requires that

1 $$\begin{aligned} \int _C{\varvec{m}}\cdot \,d{\varvec{r}} = 0 \end{aligned}$$

for every closed loop *C*. This identity constraints the number of inward intersections of the membrane into *C* to be equal to the number of outward intersections. In regions where *m* is differentiable, this requires

2 $$\begin{aligned} \varvec{\nabla }\times m = 0, \end{aligned}$$

i.e., that the director field be curl-free. Across a surface of discontinuity we must have

3 $$\begin{aligned} \text{ [[m]] }\times \nu = 0, \end{aligned}$$

where $\nu$ is the normal to the discontinuity surface. In particular, at the boundary of a rigid container $\Omega$ this condition requires

4 $$\begin{aligned} m\times \nu = 0, \end{aligned}$$

where $\nu$ is the outward unit normal to $\partial \Omega$. Let $\kappa _1$ and $\kappa _2$ be the principal curvatures of the membrane. Then, a standard identity gives

5 $$\begin{aligned} \kappa ^2 \equiv \kappa _1^2 + \kappa _2^2 = \left| (I-m\otimes m)\varvec{\nabla }m \right| ^2, \end{aligned}$$

where in components, $\left[ (I-m\otimes m)\varvec{\nabla }m \right] _{ij} = m_{i,j}-m_{i,k}m_km_j$ and $|a|^2=a_{ij}a_{ij}$.

In addition, we shall assume that the deformations of the membrane are area preserving, and that the rheology of the membrane is capable of relaxing all in plane shears. Assuming for simplicity that the strain energy density *W* per unit area of membrane bears a power law dependence on $\kappa$ gives

6 $$\begin{aligned} W^{\text {ben}}(m) = \sigma B\left| (I-m\otimes m)\varvec{\nabla }m \right| ^p, \end{aligned}$$

where $p\ge 1$ and *B* is the bending stiffness of the membrane. For $p=2$, (6) reduces to the classical Canham bending energy [24]. We note that in the absence of spontaneous curvature, the energy further reduces (modulo boundary terms) to the Helfrich bending energy [25] by an application of the Gauss–Bonnet theorem [26]. Finally, the total free energy of the membrane is

7 $$\begin{aligned} E(m) = \int _{\Omega } \sigma B \left| (I-m\otimes m)\varvec{\nabla }m \right| ^p \, dx, \end{aligned}$$

where $\Omega$ is the domain of the container enclosing the membrane. We assume that the configuration adopted by dense membrane packings are, or are close to, energy minimizers. Therefore, the optimal packaging arrangement of the membrane follows from the minimization problem

8a $$\begin{aligned} \text {inf} \,&E(m) \end{aligned}$$

8b $$\begin{aligned} \text {subject to:} \quad \nabla \times m&= 0, \quad \text {in}\,\Omega \end{aligned}$$

8c $$\begin{aligned} |m|&= \sigma , \quad \text {in} \, \Omega \end{aligned}$$

8d $$\begin{aligned} m \times \nu&= 0, \quad \text {on}\, \partial \Omega . \end{aligned}$$

An alternative formulation is obtained by enforcing the curl constraint (8b) through the introduction of a scalar potential, i.e., by setting

9 $$\begin{aligned} m = \nabla u. \end{aligned}$$

Differentiating the modulus constraint (8c), we obtain

10 $$\begin{aligned} 0 = m_jm_{j,i} = u_{,j}u_{,ji} = m_{i,j}m_j \end{aligned}$$

at points where *m* is differentiable. Then, (7) reduces to

11 $$\begin{aligned} E(u) = \int _{\Omega }\sigma B \left| \nabla \nabla u\right| ^p\, dx, \end{aligned}$$

where, in components, $\left| \nabla \nabla u\right| ^2 = u_{,ij}u_{,ij}$. Using these identities, problem (8a)–(8d) may be recast in the form

12a $$\begin{aligned} \text {inf} \quad&E(u) \end{aligned}$$

12b $$\begin{aligned} \text {subject to:} \quad |\nabla u|&= \sigma , \quad \text {in}\,\Omega \end{aligned}$$

12c $$\begin{aligned} u&= 0 \quad \text {on}\,\partial \Omega . \end{aligned}$$

## Finite-difference discretization

We seek to approximate solutions of problem (12) by recourse to a simple finite-difference discretization on a Cartesian point-lattice

13 $$\begin{aligned} x_h(l) = l \, h, \end{aligned}$$

where $h> 0$ is the lattice parameter and $l=(l_1,\dots ,l_n)$ are integer lattice coordinates in dimension *n* and in the index set

14 $$\begin{aligned} I_h = \{ l \,: \, x_h(l) \in \Omega \}. \end{aligned}$$

We further denote by $u_h(l)$ the value of the scalar potential at $x_h(l)$. The discretized form of the total free energy of the membrane (11) is, then,

15 $$\begin{aligned} E_h(u_h) = \sum _{l \in \text {int} I_h} W^{\text {ben}}(l) \, h^n, \end{aligned}$$

where $\text {int} I_h$ is an interior index set defined as

16 $$\begin{aligned} \text {int} I_h = \{ l \in I_h \,: \, \text {all neighboring sites} \in I_h \}, \end{aligned}$$

and $W^{\text {ben}}(l)$ denotes the strain energy density (6) at lattice site *l*, computed from the discrete scalar potential $u_h$ and second-order derivatives thereof approximated using a central-difference stencil. The modulus constraint (12b) is enforced via the penalty function

17 $$\begin{aligned} P_h(u_h) = \frac{1}{\epsilon _h} \sum _{l \in \text {int} I_h} \left( |\nabla _h u_h(l)| - \sigma \right) ^2, \end{aligned}$$

where $\nabla _h u_h(l)$ is computed by means of three-point numerical differentiation and $\epsilon _h \rightarrow 0$ is a sequence of penalty parameters. The resulting penalized energy function to be minimized is, then,

18 $$\begin{aligned} F_h(u_h) = E_h(u_h) + P_h(u_h). \end{aligned}$$

Finally, the boundary condition (12c) is enforced by setting

19 $$\begin{aligned} u_h(l) = 0, \quad l \in \partial I_h, \end{aligned}$$

over the boundary lattice sites $\partial I_h = I_h \backslash \text {int} I_h$.

**Fig. 2:**
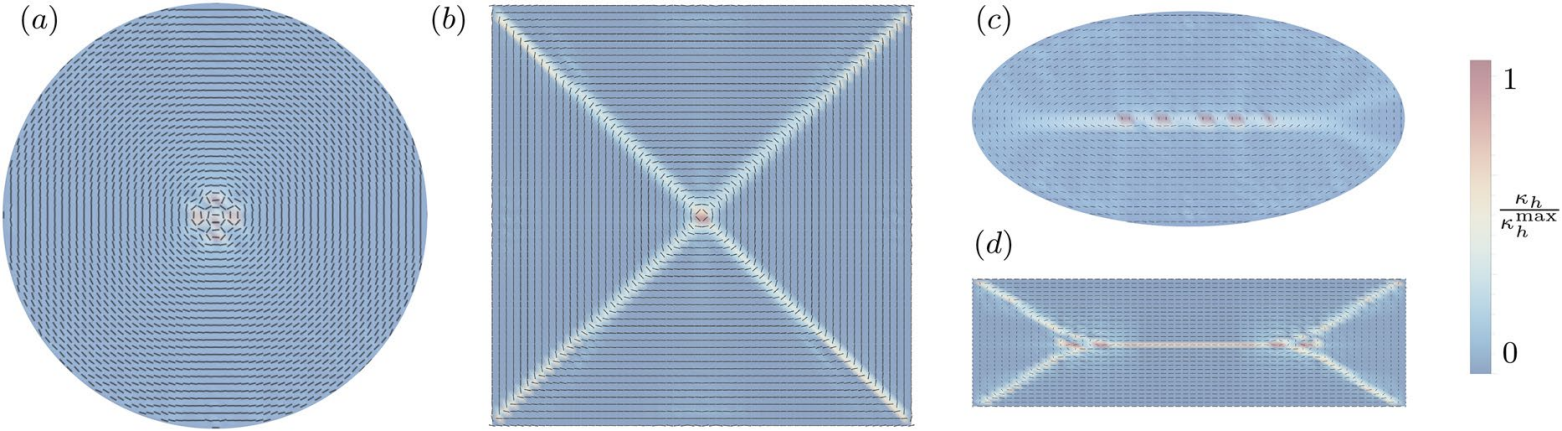
Packed membrane configurations in a spherical (**a**), square (**b**), ellipsoidal (**c**) and rectangular (**d**) confinement. Background density highlighting mean curvature scaled to the maximum curvature within each configuration. For all types of confinement, solutions comprise multiple closed surfaces

**Fig. 3:**
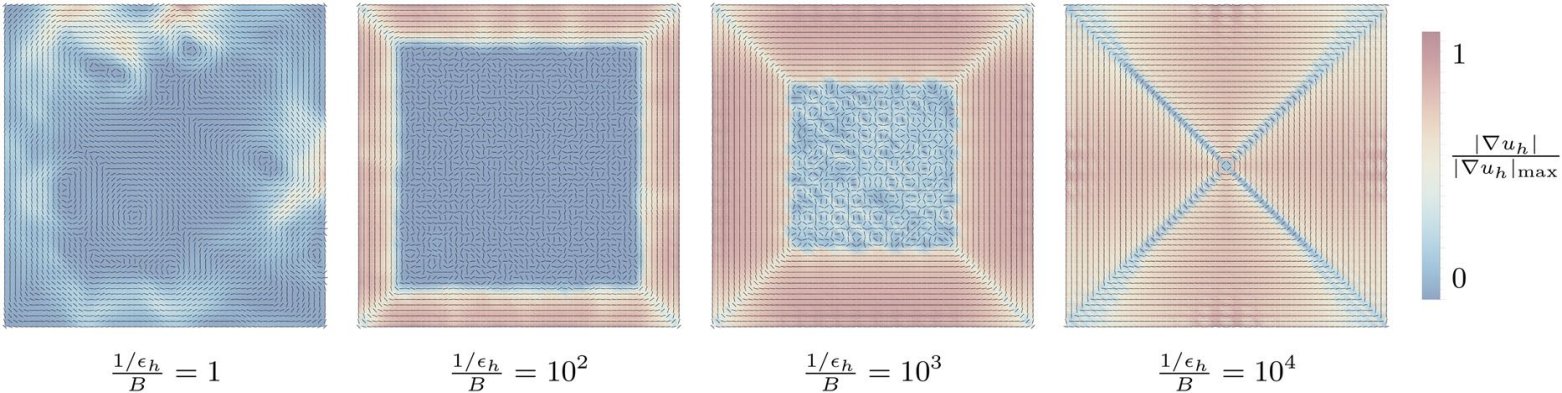
Folded membrane configurations for increasing modulus constraint, yielding fully packed membranes. Background density highlighting local membrane density scaled to the maximum density within each configuration

Numerically-computed energy-minimizing membrane configurations are shown in Figs. 2 and 3. Figure 3 illustrates the competition between bending- and modulus constraint. Figure 2 (setting $p=2$, $\sigma =1$ and $\frac{1/\epsilon _h}{B} = 10^3$) shows foliations for several selected domains. As may be seen from the results, the folding configuration follows closely a maximal solution of the eikonal equation 12b–12c in analogy to domain patterns in micromagnetics [27] and wrinkling patterns in thin films [28–30]. In all cases, the packing configuration consists of a foliation of closed surfaces stacked next to each other and filling the entire domain tangentially to its boundary. While these solutions locally satisfy the continuity constraint (1) in a weak sense (sheets are locally continuous), it is also of interest to characterize packing configurations involving single sheets. To this end, we append topological constraints to our model, as discussed next.

## Topological constraints

**Fig. 4:**
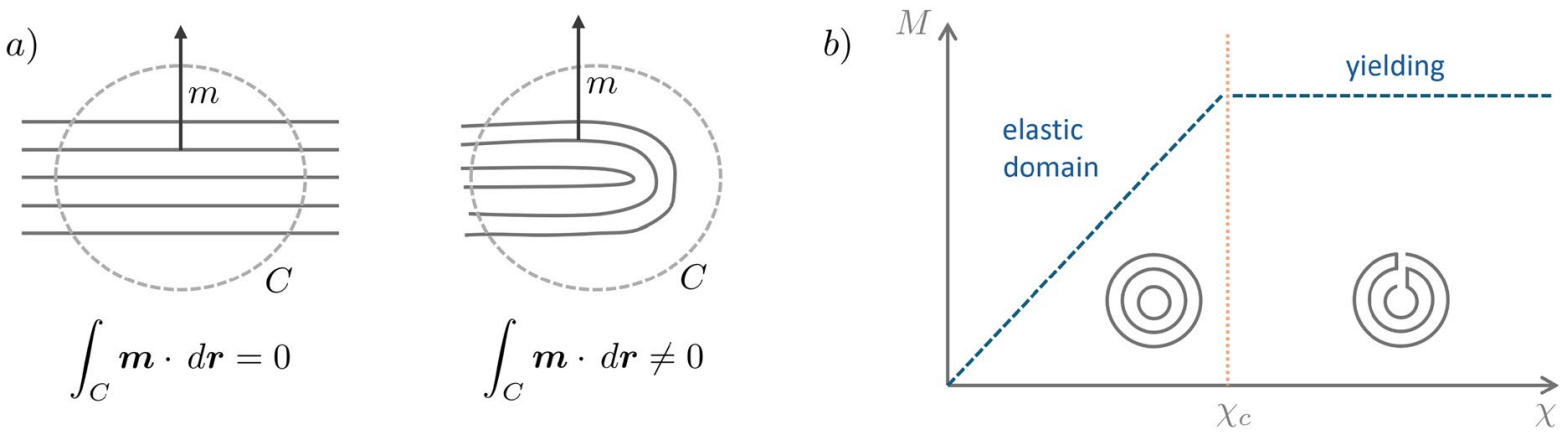
**a** Creases introduce topological singularities which fail to satisfy the continuity constraint (2). **b** Moment-curvature relation featuring an initial linear domain (pure bending), followed by yielding after a critical value $\chi _c$ is reached (development of creases)

Suppose that, in contrast to the topologically unconstrained packing configurations computed in the foregoing, we append the constraint that the membrane consist of a single sheet, with or without boundary. In order to build in this constraint, we begin by assuming that the membrane can develop sharp *creases*, which are topological singularities where the constraint fails (2) (see sketch in Fig. 4a). In order to accommodate the singularities, we enforce (2) by means of the extended energy

20a $$\begin{aligned} \text {inf} \,&\left( E(m) + \lambda \int _\Omega |\nabla \times m|\,dx\right) \end{aligned}$$

20b $$\begin{aligned} \text {subject to:} \quad |m|&= \sigma , \quad \text {in} \, \Omega \end{aligned}$$

20c $$\begin{aligned} m \times \nu&= 0, \quad \text {on}\, \partial \Omega . \end{aligned}$$

where $\lambda$ is the energy per unit length of crease. The energy (20) also follows as a sharp-singularity approximation of the energy density

21 $$\begin{aligned} \begin{aligned}&W^{\text {ben}}(m,\nabla m) = \\ &\left\{ \begin{array}{ll} \sigma B\left| \chi \right| ^2, & \text {if } \left| \chi \right| \le \chi _c,\\ \sigma B ( 2 \left| \chi \right| - 2 \chi _c + \chi _c^2 ), & \text {otherwise}, \end{array} \right. \end{aligned} \end{aligned}$$

where we write

22 $$\begin{aligned} \chi = (I-m\otimes m) \, \nabla m. \end{aligned}$$

In view of the linear growth of this energy density, sharp creases have finite energy per unit length

23 $$\begin{aligned} \lambda = 2 \pi \sigma B. \end{aligned}$$

This model is in analogy to the classical deformation theory of plasticity recast in terms of moment/curvature variables. As sketched in Fig. 4b, the moment-curvature relation $M=DW^{\text {ben}}(\chi )$ has an initial linear $M-\chi$ dependence up to $\chi _c$ (or elastic domain), followed by yielding at constant *M* (development of creases).

**Fig. 5:**
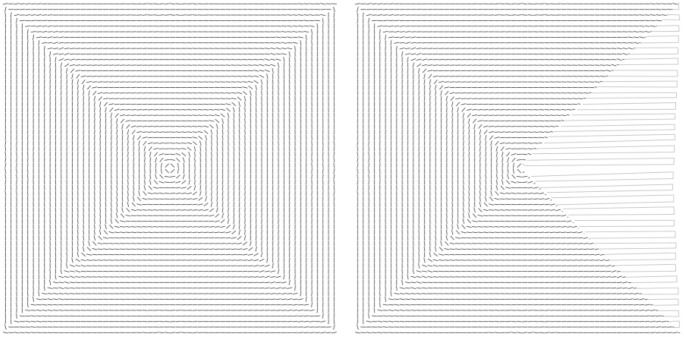
A posteriori clipping construction applied to foliations in a square confinement. Left: original foliation. Right: crease construction

For this choice of energy, the solutions in the preceding section are still valid if we assume that the magnitude $|\chi |$ of the bending strain is everywhere less than $\chi _c$. Taking solutions of the preceding section as a starting point, the topology of the membrane can then be modified a posteriori—and a single sheet obtained—by judiciously clipping and reconnecting the membrane, albeit at an energy cost concentrated in the resulting creases. The aim of the construction is to introduce a distribution of cuts and creases a posteriori that minimizes the total crease energy. Figure 5 shows the result of this a posteriori construction applied to the solution within a square confinement in Fig. 2. Evidently, the energy-minimizing crease pattern is exceedingly non-unique.

**Fig. 6:**
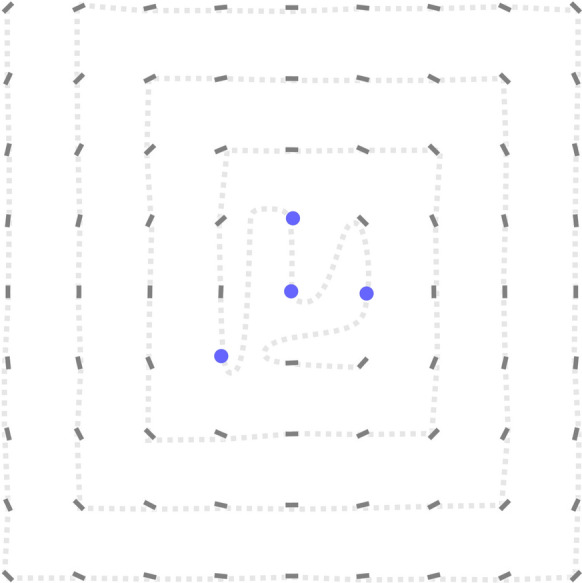
Packed membrane configuration in a square confinement with single-sheet topological constraint. Blue circles highlighting the computed location of creases. (Color figure online)

Alternatively, approximate solutions can be obtained directly by minimizing a finite-difference discretization of energy (20). The resulting solutions are shown in Fig. 6 for the case of a rectangular confinement. The blue circles mark the computed location of creases, where the number of creases is fixed to equal the total number of foliations. We observed that the initial packing is clipped at selected points and the membrane is clipped and spliced so as to generate a single sheet. At every crease, the membrane turns by an angle of $\pi$, which costs $\lambda$ in terms of energy (23).

## Discussion

We have developed a simple mathematical model to solve for optimal packing configurations of densely packed fluid membranes. Numerically computed energy minimizing configurations follow a maximal solution of the eikonal equation, consisting of a foliation of closed surfaces. While these foliations locally satisfy the continuity constraint, topological constraints may be added to obtain solutions consisting of a single sheet. We have proposed a construction that builds on foliated solutions and modifies their topology a posteriori through clipping and reconnection resulting in line singularities or *creases*. The topologically constrained solutions can also be obtained directly by allowing for—and penalizing—topological singularities.

The proposed model appears to capture the essential physics of tightly packed membranes despite its simplicity and suggests various worthwhile extensions. Thus, we do not expect single-sheet packing configurations to be unique. Therefore, it would be of interest to extend the analysis to an examination of different competitors in terms of crease distribution. In addition, the single sheet topological constraint may be augmented to also enforce single sheets with boundary (via, e.g., exploiting topological invariants such as the Euler characteristic). Finally, whereas the numerical analysis in this paper focuses on two dimensional cases, extensions to three dimensions may provide further valuable insights [31, 32].
